# Supplementary material for: Pressureless glass crystallization of transparent yttrium aluminum garnet-based nanoceramics
Source: Nat Commun. 2018 Mar 21;9:1175. doi: 10.1038/s41467-018-03467-7 (PMC5862837; doi:10.1038/s41467-018-03467-7)
Supplement: Supplementary file 1 — Supplementary Information(DOCX 8537 kb) [file 41467_2018_3467_MOESM1_ESM.docx]

**SUPPLEMENTARY INFORMATION**

**Pressureless glass crystallization of transparent yttrium aluminum garnet-based nanoceramics**

Xiaoguang Ma^1,2^†, Xiaoyu Li^1^†, Jianqiang Li^1,3*^, Cécile Genevois^4^, Bingqian Ma^1^, Auriane Etienne^5^, Chunlei Wan^6^, Emmanuel Véron^4^, Zhijian Peng^2^, Mathieu Allix^4,*^

^1^ National Engineering Laboratory for Hydrometallurgical Cleaner Production Technology, CAS Key Laboratory of Green Process and Engineering, Institute of Process Engineering, Chinese Academy of Sciences, Beijing 100190, P. R. China.

^2^ School of Engineering and Technology, China University of Geosciences, Beijing 100083, P. R. China.

^3^ School of Chemical Engineering, University of Chinese Academy of Sciences, Beijing 100049, P.R. China.

^4^ CNRS, CEMHTI UPR 3079, Univ. Orléans, F-45071 Orléans, France.

^5^ Groupe de Physique des Matériaux, CNRS, UNIROUEN, INSA Rouen, Normandie Univ, 76000 Rouen, France.

^6^ State Key Lab of New Ceramics and Fine Processing, School of Materials Science and Engineering, Tsinghua University, Beijing 100084, PR China.

*Correspondence to: E-mail address: [jqli@ipe.ac.cn](mailto:jqli@ipe.ac.cn), [allix@cnrs-orleans.fr](mailto:allix@cnrs-orleans.fr)

†These authors contributed equally to this work.

**Supplementary Figure 1**| Phase diagram of the Al_2_O_3_-Y_2_O_3_ system and enlargement of the alumina-rich region originally published in Jaroslav et al., J. Mater. Sci. 15 7 1709 (1980). This figure is not included under the CCBY license for this manuscript. © Chapman and Hall Ltd. 1980.

(d)


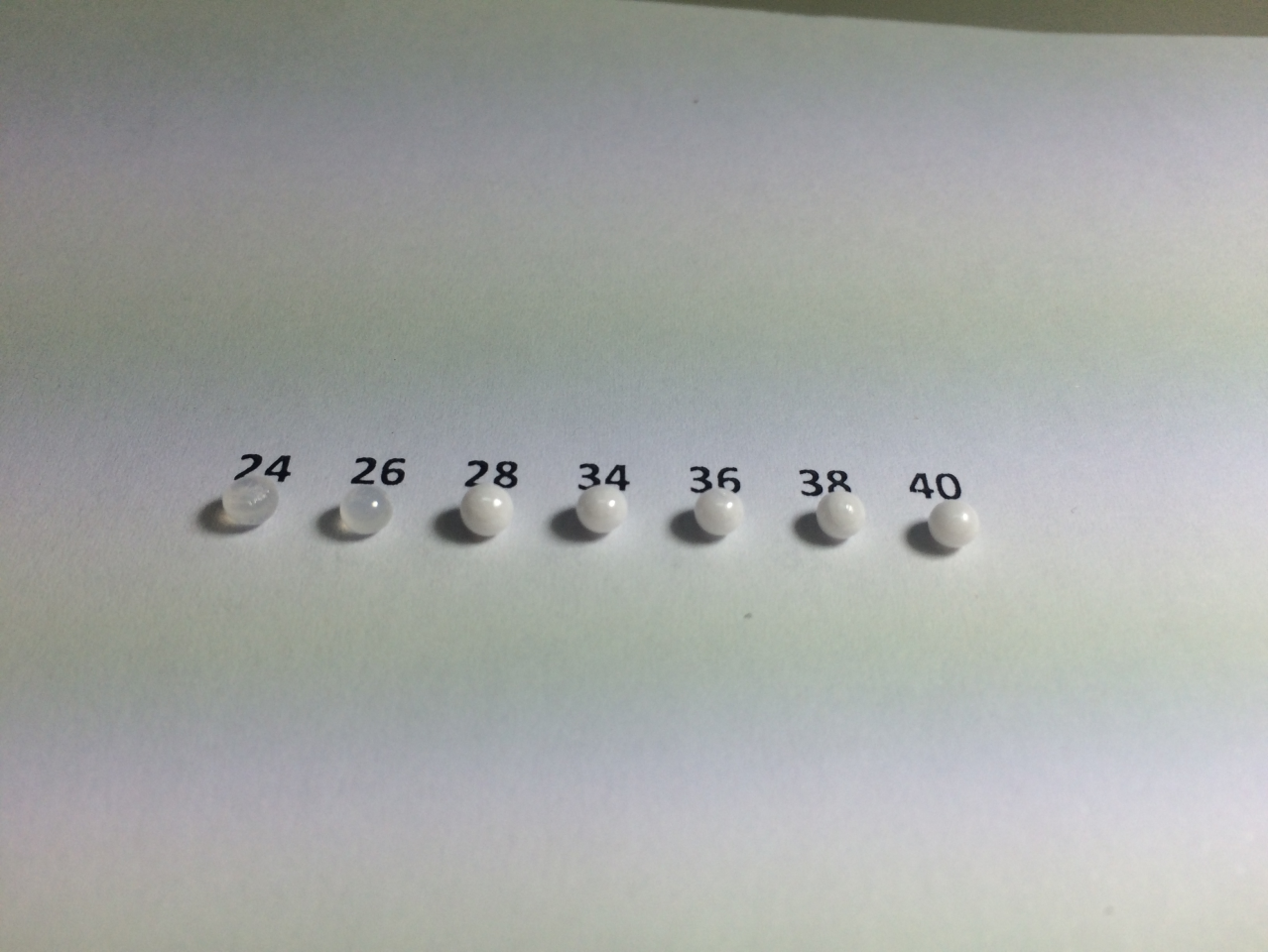


**Supplementary Figure 2| Microstructure of the AY28 (72 mol% Al_2_O_3_ - 28 mol% Y_2_O_3_) and AY26 (74 mol% Al_2_O_3_ - 26 mol% Y_2_O_3_) Y_2_O_3_-Al_2_O_3_ composite nanoceramics.** SEM images of the following nominal compositions: (a) 72 mol% Al_2_O_3_ - 28 mol% Y_2_O_3_ (AY28) biphasic ceramic (theoretical content: 81 wt% YAG and 19 wt% Al_2_O_3_) and (b) 74 mol% Al_2_O_3_ - 26 mol% Y_2_O_3_ (AY26) biphasic ceramic (theoretical content: 77 wt% YAG and 23 wt% Al_2_O_3_). The bright contrast is assigned to YAG whereas the dark phase corresponds to Al_2_O_3_. (c) Rietveld refinements from X-ray powder diffraction data collected on (up) AY28 and (down) AY26 ceramics elaborated from glasses crystallized 2h at 1100 ℃ in air. (d) The photograph of the AY24-AY40 glasses after fully crystallization.

**Supplementary Figure 3| Transparency of the AY26 (74 mol% Al_2_O_3_ - 26 mol% Y_2_O_3_) glass.** Transmittance spectrum in UV-VIS-NIR and MIR region of the YAG-Al_2_O_3_ glass measured through a 1.5 mm thick sample. The typical dashed line corresponds to the theoretical maximum transmission (87% corresponding to n=1.71 which was measured experimentally). The embedded photograph of the glass (diameter: 4 mm) is elevated 2 cm above the text.

**Supplementary Figure 4| Amorphousness of the AY26 (74 mol% Al_2_O_3_ - 26 mol% Y_2_O_3_) glass.** X-ray powder diffractogram of the AY26 glass precursor. The embedded photograph of the sample (4 mm diameter) is elevated 2 cm above the text.


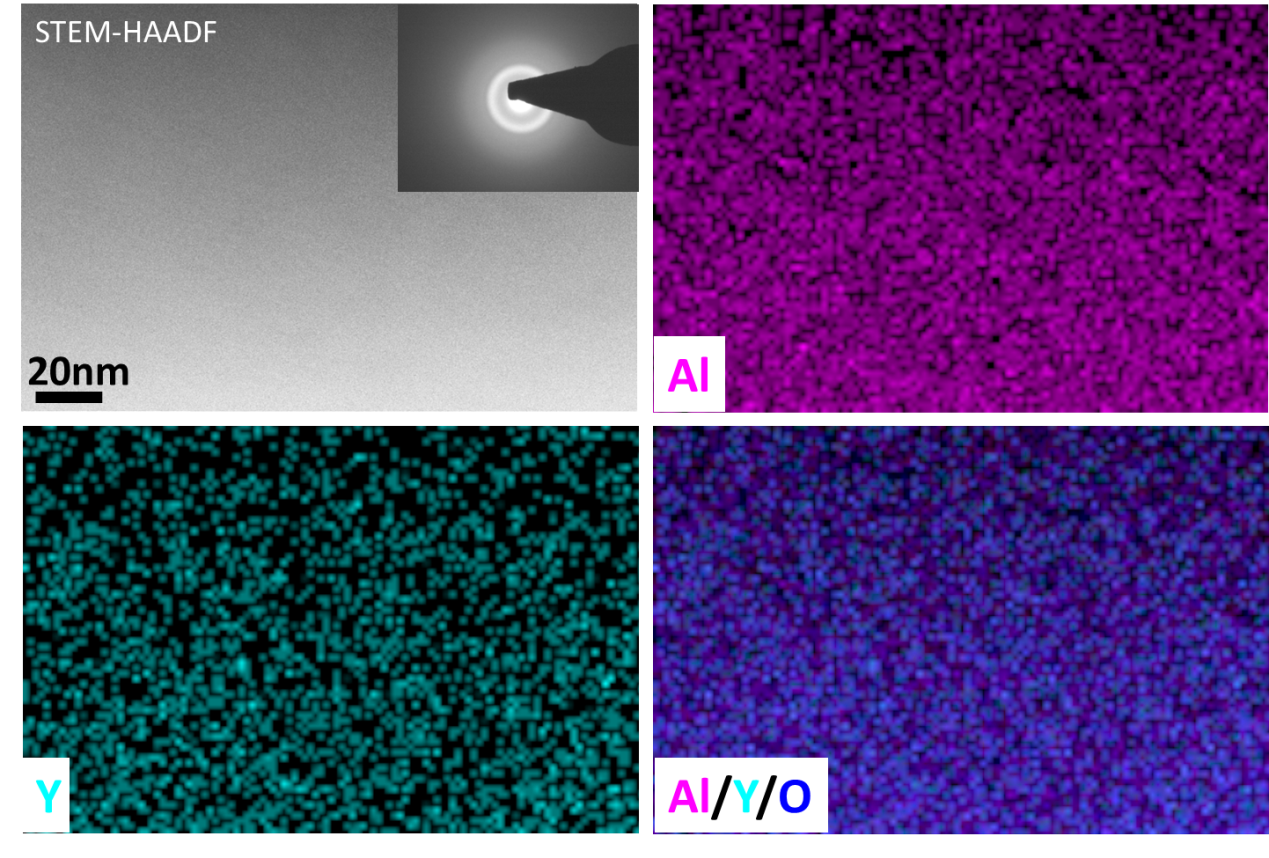


**Supplementary Figure 5| Nanoscale chemical homogeneity of the AY26 (74 mol% Al_2_O_3_ - 26 mol% Y_2_O_3_) glass material.** (a) STEM-HAADF image of the AY26 parent glass. The corresponding selected area electron diffraction (SAED) pattern is embedded. The associated Al (purple), Y (cyan) O (blue) STEM-EDX elemental maps show chemical homogeneity of the glass.

**Supplementary Figure 6| Transparency of the AY26 (74 mol% Al_2_O_3_ - 26 mol% Y_2_O_3_) glass and transparent YAG-Al_2_O_3_ biphasic ceramics synthesized at different temperatures.** Transmittance spectra measured through 1.5 mm thick samples in UV-VIS-NIR and MIR region of the AY26 YAG-Al_2_O_3_ glass and ceramics crystallized at various temperatures. The dashed line corresponds to the theoretical maximum transmission (87%) of the AY26 glass which was approximated as constant for simplicity here. The photographs of the glass and ceramic materials (4 mm diameter) are embedded.

**Supplementary Figure 7| HRTEM micrograph of the YAG-Al_2_O_3_ composite ceramic with corresponding FFT and simulated ED patterns.** The YAG nanocrystals are surrounded by thin Al_2_O_3_ crystalline areas assigned to γ-Al_2_O_3_. Light grey arrows on YAG-1 FFT pattern denote forbidden reflections showing up from double diffraction effect.

**Supplementary Figure 8| Density evolution of the AY26 (74 mol% Al_2_O_3_ - 26 mol% Y_2_O_3_) YAG-Al_2_O_3_ material as a function of the crystallization temperature.** Starting from glass (d = 3.80 g.cm^-3^), the density increases during crystallization and still evolves with the nature of the Al_2_O_3_ polymorph. For comparison, crystalline YAG exhibits a 4.55 g.cm^-3^ density^1^.

**Supplementary Figure 9| Transparency of YAG single crystal, YAG transparent ceramic (SICCAS), YAG-Al_2_O_3_ composite nanoceramic (crystallized from AY26 glass at 1100°C for 2h) and YAG-Al_2_O_3_ (AY26) glass.** The transmittance spectra were measured through 1.5 mm thick samples in UV-VIS-NIR and MIR region. The absorption band located around 3 μm is attributed to the absorption of free hydroxyl (OH) group, which is commonly observed in oxide glasses^2^.

**Supplementary Figure 10| SEM pattern of the microstructure of the commercial transparent YAG ceramic provided by the Shanghai Institute of Ceramics, Chinese Academy of Sciences (SICCAS) laboratory.** The average crystal size is 30 microns. In order to reveal the grain boundaries, the YAG ceramic was thermally etched at 1200 °C for 2h.

**Supplementary Figure 11| Thermal conductivity of YAG-Al_2_O_3_ composite nanoceramics.** (a) Experimental thermal diffusivity of the YAG-Al_2_O_3_ nanoceramic crystallized at 1100°C for 2h, YAG single crystal and commercial YAG transparent ceramic (SICCAS). (b) Heat capacity of YAG and YAG-Al_2_O_3_ nanoceramic crystallized at 1100°C for 2h. (c) Thermal conductivity at different temperatures of the YAG-Al_2_O_3_ nanoceramic synthesized at 1100°C for 2h, YAG single crystal and YAG transparent ceramic (error bars determined based on the results from 3 tests). (d) The thermal conductivity values at room temperature of the YAG-Al_2_O_3_ nanoceramics, the YAG single crystal and the commercial YAG transparent ceramic are presented in comparison to several YAG-based materials: YAG crystals dispersed in epoxy resin, YAG glass-ceramic and the AY26 YAG-Al_2_O_3_ glass^3, 4^. Large colored areas are presented in order to take into account the experimental error (estimated on the deviation observed for the YAG transparent ceramic and YAG single crystal materials) on the thermal conductivity measurement related to the small size of the measured samples. The thermal conductivity of the YAG-Al_2_O_3_ nanoceramics synthesized at different temperatures show a dependence of the crystallization temperature, therefore evidencing relation with the materials grain size (interface thermal resistance).

**Supplementary References**

1. Paradis, P. F., Yua, J., Ishikawa, T., Aoyama, T., Yoda, S. & Weber, J.K.R. Contactless density measurement of superheated and undercooled liquid Y_3_Al_5_O_12_. *J. Cryst. Growth.* 249, 523-530 (2003)

2. Abe, Y. & Clark, D. E. Determination of combined water in glasses by infrared spectroscopy. *J. Mater. Sci. Lett.* 9, 244-245 (1990)

3. Chen, D. Q. & Chen, Y. Transparent Ce^3+^: Y_3_Al_5_O_12_ glass ceramic for organic-resin-free white-light-emitting diodes. *Ceram. Int.* 40, 15325–15329 (2014)

4. Zhang, R., Lin, H., Yu, Y. L., Chen, D. Q., Xu, J. & Wang, Y. S. A new-generation color converter for high-power white LED: transparent Ce^3+^:YAG phosphor-in-glass. *Laser Photonics Rev.* 1, 158-164 (2014)
